# Evaluating recovery potential of the northern white rhinoceros from cryopreserved somatic cells

**DOI:** 10.1101/gr.227603.117

**Published:** 2018-06

**Authors:** Tate Tunstall, Richard Kock, Jiri Vahala, Mark Diekhans, Ian Fiddes, Joel Armstrong, Benedict Paten, Oliver A. Ryder, Cynthia C. Steiner

**Affiliations:** 1 San Diego Zoo Institute for Conservation Research, Escondido, California 92027, USA;; 2 Royal Veterinary College, University of London, London NW1 0TU, United Kingdom;; 3 Dvur Krlov Zoo, Dvr Krlov nad Labem 544 01, Czech Republic;; 4 Jack Baskin School of Engineering, University California Santa Cruz, Santa Cruz, California 95064, USA

## Abstract

The critically endangered northern white rhinoceros is believed to be extinct in the wild, with the recent death of the last male leaving only two remaining individuals in captivity. Its extinction would appear inevitable, but the development of advanced cell and reproductive technologies such as cloning by nuclear transfer and the artificial production of gametes via stem cells differentiation offer a second chance for its survival. In this work, we analyzed genome-wide levels of genetic diversity, inbreeding, population history, and demography of the white rhinoceros sequenced from cryopreserved somatic cells, with the goal of informing how genetically valuable individuals could be used in future efforts toward the genetic rescue of the northern white rhinoceros. We present the first sequenced genomes of the northern white rhinoceros, which show relatively high levels of heterozygosity and an average genetic divergence of 0.1% compared with the southern subspecies. The two white rhinoceros subspecies appear to be closely related, with low genetic admixture and a divergent time <80,000 yr ago. Inbreeding, as measured by runs of homozygosity, appears slightly higher in the southern than the northern white rhinoceros. This work demonstrates the value of the northern white rhinoceros cryopreserved genetic material as a potential gene pool for saving this subspecies from extinction.

The worldwide loss of wildlife has been described as the sixth mass extinction (Wake and Vredenburg 2008; Kolbert 2014), with 22% of mammals at risk of extinction (http://www.iucnredlist.org). Rhinoceroses as a group are particularly affected, with three of the five extant species listed as critically endangered (Javan, Sumatran, and black rhinoceroses), one listed as vulnerable (greater one-horned rhinoceros), and only one, the white rhinoceros (*Ceratotherium simum*), listed as near threatened. Despite continuing threats, including poaching and habitat destruction, the southern population of white rhinoceros known as the southern white rhinoceros (SWR; *C. simum simum*) is currently the most abundant rhinoceros in the world, with about 20,000 individuals living primarily in South Africa (Emslie 2012). Thought to be nearly extinct at the beginning of the 20th century after being reduced to a single population of 20–50 individuals (Emslie 2012), subsequent conservation efforts have led to a dramatic recovery of this subspecies. However, poaching remains a serious threat, and white rhinoceroses are killed at a rate of about two per day, primarily to harvest their horns (Emslie and Knight 2014).

The northern population of white rhinoceros or northern white rhinoceros (NWR; *C. simum cottoni*) is listed as critically endangered and is believed to be extinct in the wild, with the recent death of the last male, Sudan, leaving only two remaining individuals in captivity (Emslie 2012). Extensive poaching and civil war throughout the 20th century has led to the near extinction of this subspecies, which occurred in the Central African Republic, Chad, the Democratic Republic of the Congo, South Sudan, Sudan, and Uganda (Fig. 1A; Rookmaaker and Antoine 2012). The last live wild NWR was seen in 2006, and extensive foot surveys have not discovered any remaining individuals (Emslie 2012).

**Figure 1.**
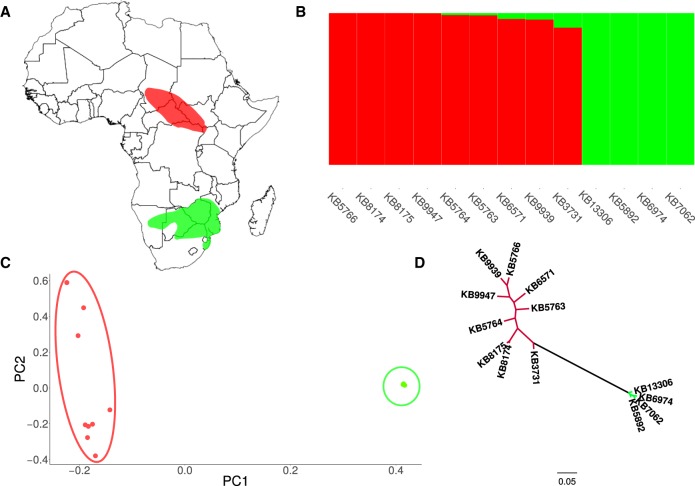
White rhinoceros distribution, population structure, and phylogeny. (*A*) Historical distributions of the northern (red) and southern white rhinoceros (green) in Africa according to work by Rookmaaker and Antoine (2012). (*B*) ADMIXTURE results of the nine northern and four southern white rhinoceroses. All individuals were grouped in two clusters (*K* = 2) colored by population. (*C*) PCA, with PC1 explaining 35% of the variance, and PC2, which explained 10%. (*D*) Maximum likelihood tree of the relationship between northern and southern white rhinoceroses using genome-wide SNPs.

The recovery of this critically endangered subspecies faces numerous challenges. The remaining two NWR females are not suitable for reproduction, and concerns still persist for the safety of these animals in the wild due to poaching. Saragusty et al. (2016) identified the steps required for a long-term program to “rewind” the extinction process and secure a viable population with sustainable levels of genetic diversity. These steps include estimating the genetic divergence of northern and southern white rhinoceros populations, validating their taxonomic status as subspecies, and assessing the genetic variation in the NWR through genome-wide comparisons with the SWR.

Traditional in situ and ex situ conservation efforts, such as the establishment of a captive breeding program and antipoaching measures, have not been effective in saving the NWR from the brink of extinction. The last NWR calf born in captivity was in 2000 (Saragusty et al. 2016), and efforts at captive breeding have not been successful due to limited reproduction in both the wild-captured founder animals as well as those born in captivity. The fate of the NWR would appear to be sealed, but the development of advanced cell and reproductive technologies, such as cloning by nuclear transfer and the artificial production of gametes via stem cells differentiation, offers a possible path forward (Nayernia et al. 2006; Hayashi and Saitou 2013; Easley et al. 2015; Hendriks et al. 2015; Saragusty et al. 2016). These reproductive technologies could provide new tools for the rescue of endangered, wild populations, especially those from where samples have been collected before large declines in genetic diversity. Populations with low genetic diversity can face increased susceptibility to disease (Tasmanian devils) (Morris et al. 2013) and reduced fertility (Felidae [Neubauer et al. 2004]; Iberian lynx [Ruiz-López et al. 2012]); therefore, banking of genetic material in the form of cells and gametes is crucial for future genetic rescue efforts.

Over the past 30 yr, the San Diego Zoo Frozen Zoo has cultured and banked 12 NWR fibroblast cell lines representing eight presumably unrelated founders. These cells correspond to the remaining living genetic material of the NWR, and as proposed by Saragusty et al. (2016), could be used for its genetic rescue. Of course, this approach would be a difficult undertaking, but success could greatly expand the genetic pool of this subspecies beyond the last two remaining individuals and help to develop advanced genetic and reproductive technologies that could also benefit other rhinoceros species in peril. Large-scale habitat destruction has not been the reason for the extirpation of the NWR (Saragusty et al. 2016), so presumably there is potential habitat available for its reintroduction. The greatest obstacles to the successful recovery of the NWR is the continuing threat of poaching in the wild and its small population size.

In this work, we present the complete genome sequences of four SWRs and nine NWRs derived from cryobanked material and provide information on genome-wide levels of genetic diversity and inbreeding among NWRs with the goal of assisting informed-based decisions on genetically valuable material to be used in future efforts of genetic rescue and assisted reproduction of this subspecies. By using genomic data, we inferred the recent population history and demography of the two white rhinoceros subspecies and identified potential signatures of selection in the NWR that may suggest local adaptation.

## Results

We sequenced the genomes of nine NWR and four SWR individuals (Table 1). All sequences were generated using Illumina short-read sequencing, and were aligned to the reference SWR genome (cerSim1), which is assembled to the scaffold level with an N50 of 26 Mbp. After calling variants and filtering, about 9.4 million single-nucleotide polymorphisms (SNPs) were identified; 1,723,931 SNPs were polymorphic in both subspecies, 1,869,825 were fixed in both, and 2,511,658 and 4,065,345 were unique SNP variants in the SWR and the NWR, respectively (Supplemental Fig. S2). The average coverage depth of the NWR and SWR genomes sequenced ranged from 10× to 15×.

**Table 1.**
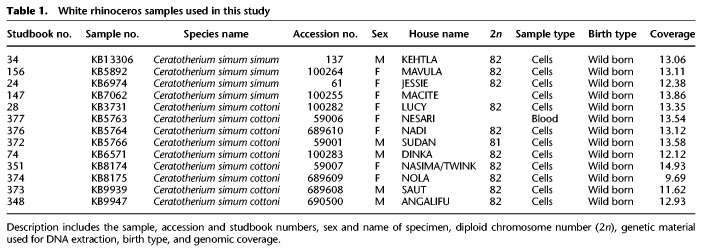
White rhinoceros samples used in this study

To examine population structure and admixture in the white rhinoceros, we used ADMIXTURE (Alexander et al. 2009) and EIGENSTRAT (Price et al. 2006) analyses on a marker set of approximately 144,000 SNPs that had been thinned for linkage disequilibrium. Results from ADMIXTURE suggest that the NWR and the SWR subspecies represent two distinct populations with little genetic admixture (Fig. 1B, 10-fold cross validation in Supplemental Fig. S3). These results may reflect historical gene flow between populations but most likely reflect the effect of low level of genetic differentiation between subspecies, homoplasy, or incomplete lineage sorting. Principal component analysis (PCA) also supported the two populations as distinct clusters (Fig. 1C). PC1 (35% of the variance) separates the rhinoceroses into two distinct populations, whereas PC2 (10% of the variance) evidences genetic variation in the NWR. By using the same SNP data, we generated a maximum likelihood tree using SNPhylo (Lee et al. 2014) that showed strong support for two distinct northern and southern clades (100%, in 100 bootstrapped samples) (Fig. 1D) but weaker support for any within-subspecies phylogenetic relationships. Similar phylogenetic results were obtained after comparing complete mitochondrial genomes from both subspecies (Supplemental Fig. S4).

**Figure 2.**
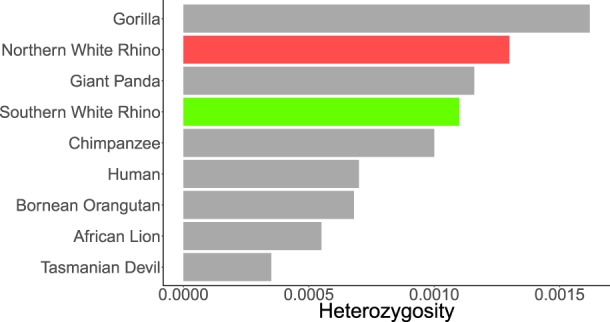
Estimates of genome-wide diversity in the northern (red) and southern (green) white rhinoceros relative to other mammalian genomes. Heterozygosity in each species is based on work by Dobrynin et al. (2015).

As a measure of genetic variation, we estimated genome-wide heterozygosity calculated as the proportion of heterozygous sites in the genome of each individual. We found that the mean genome-wide heterozygosity in the NWR was slightly higher (0.0011) than that of the SWR samples studied (0.0009). Both subspecies had relatively high levels of genetic variation compared with other threatened species such as the Tasmanian devil or the Bornean orangutan (Fig. 2). We also found an average total genetic divergence of 0.001 (0.1%) between the two subspecies, which is similar to values calculated for other mammalian subspecies (see Supplemental Table S1).

**Figure 3.**
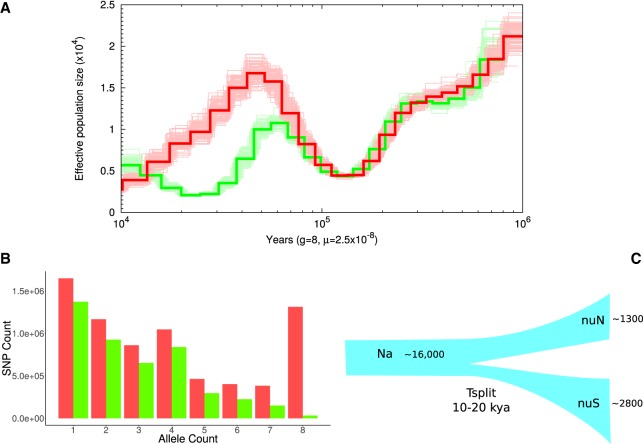
Demographic history and divergence time of the white rhinoceros. (*A*) PSMC plot for one northern (KB8174; red) and one southern (KB7062; green) white rhinoceros genomes with highest coverage (15× and 14×, respectively), assuming a generation time (g) of 8 yr, a mutation rate (µ) of 2.5 × 10^−8^, and a false-negative rate of 6% due to low coverage. Lighter lines represent 100 bootstrap samples. (*B*) Site-frequency spectra of the four northern (KB3731, KB5763, KB6571, KB8174) and four southern white rhinoceroses used for the ∂a∂i analysis, showing the number of SNPs with the respective allele count in each subspecies. (*C*) The best-supported ∂a∂i model showing divergence of the two rhinoceros populations with low rates of migration and population growth. Ancestral effective population size (Na), current effective population sizes for the northern (nuN) and southern (nuS) white rhinoceros, and the split of both populations (Tsplit) are shown.

To infer the divergence time and demographic history of the northern and southern subspecies, we used pairwise sequentially Markovian coalescent (PSMC) (Fig. 3A; Li and Durbin 2011). Both populations initially appear to share a similar demographic history, undergoing a relatively ancient decline in population size ∼800,000 yr ago (kya), followed by population recovery starting ∼100 kya. The white rhinoceros then split into two populations ∼80 kya with variable demographic trajectories. The NWR increases in effective population size, followed by a decline, while the SWR declines in effective population size, followed by a recovery to a larger size than the NWR. The NWR population seems to have reached a maximum effective population size of 15,000 individuals ∼40 kya, while the SWR reached a maximum effective population size of 10,000 individuals ∼50 kya.

To further explore demographic scenarios, we also tested a series of increasingly complex demographic models using ∂a∂i (Gutenkunst et al. 2009). In each model, the two populations split either into a fraction of the ancestral population size or into two distinct populations and then grew or shrank to the current population sizes. The most well supported model proposes a split of the NWR and SWR populations into a fraction of the ancestral population, followed by a change in effective population size to the recent numbers (see Supplemental Table S3). We assumed a mutation rate of 2.5 × 10^−8^, which has been estimated for humans (Nachman and Crowell 2000), and a generation time of 8 yr (Hillman-Smith et al. 1986). This model estimated a divergence time of ∼10–20 kya for the two white rhinoceros populations, which is lower than the 80 kya estimate from the PSMC analysis. Our model showed that both populations split from an ancestral effective population of 16,000 individuals, followed by growth in both the NWR and SWR. Current effective population size estimates correspond to 1300 and 2800 for the NWR and SWR, respectively (Fig. 3C).

**Figure 4.**
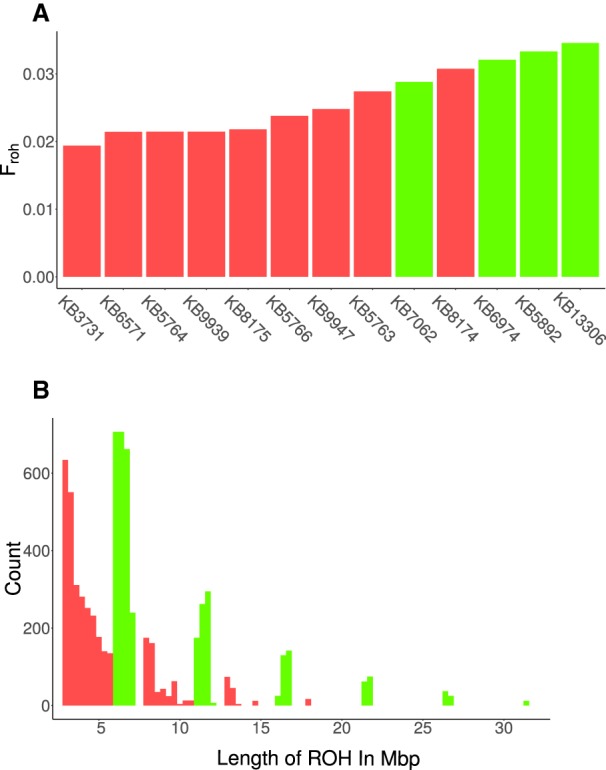
Inbreeding in the white rhinoceros. (*A*) Estimated percentage of genome-wide autozygosity (*F*_roh_) for nine northern (red) and four southern (green) white rhinoceroses. (*B*) Length distribution of ROH in each NWR and SWR (individual order from *left* to *right*: KB8174, KB5763, KB5766, KB133068, KB8175, KB3731, KB5764, KB6571, KB9939, KB13306, KB6974, KB5892, KB7062), grouped by ROH lengths from 5 to 30 Mbp.

To determine inbreeding in white rhinoceroses, we calculated runs of homozygosity (ROH) by scanning 1-Mbp sliding windows throughout the genomes. Our analysis showed similar levels of autozygosity (*F*_roh_) in the two white rhinoceros subspecies, with estimates of *F*_roh_ slightly higher in the southern subspecies overall (Fig. 4A). Among the NWRs examined, all individuals except one (KB8174) showed the lower levels of inbreeding, with between 2% and 3% of their genome being autozygous; in contrast, three of the four SWR showed autozygosity >3% (KB6974, KB5892, and KB13306). Additionally, both the NWR and the SWR had a median ROH length of 1.6 Mbp, but the SWR had a larger maximum ROH length of 32 Mbp versus 23 Mbp in the NWR (Fig. 4B; Supplemental Fig. S5), which suggests that the differences in autozygosity may be due to recent inbreeding in the SWR. These estimators, *F*_roh_ and the low mean size of ROH, in addition to the number of unique SNPs per individual (see Supplemental Table S2), allowed us to identify NWR cell lines particularly valuable for any attempt of genetic rescue. These samples include individuals KB3731, KB9939, KB6571, and KB5764, which have low levels of autozygosity, a low mean size of ROH, and a high number of unique SNPs.

To identify regions in the NWR genome potentially under selection, we calculated Tajima's D in sliding windows of 50 kbp and identified regions where the calculated values fell within the 1% quantiles as potentially under selection (Supplemental Fig. S6). We identified all coding SNPs that occurred in these regions according to the generated white rhinoceros genome annotation. This resulted in about 240 SNPs in 28 genes potentially identified under selection (Supplemental Table S4). Among those, we found 100 SNPs that were fixed differences in either subspecies. None of the SNPs were fixed in both subspecies, suggesting soft selective sweeps. We then identified the gene ontology categories for each gene using Ensembl, finding the olfactory receptor genes as the most common category, with 11 genes involved in sensory perception of smell potentially under selection (Table 2).

**Table 2.**
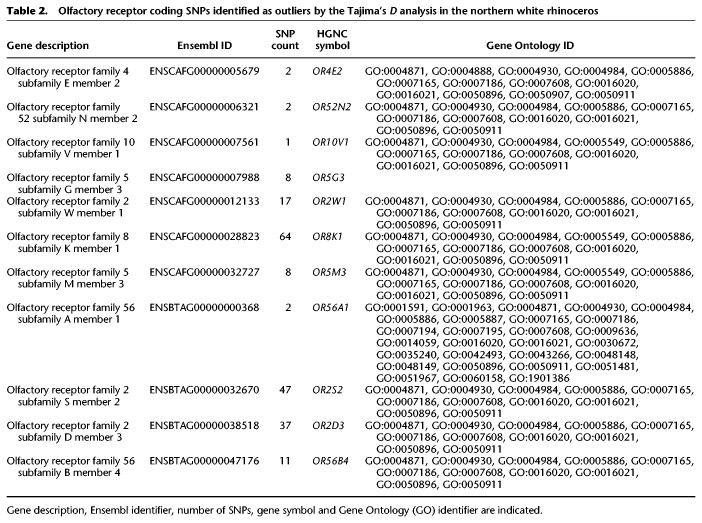
Olfactory receptor coding SNPs identified as outliers by the Tajima's *D* analysis in the northern white rhinoceros

## Discussion

Whole-genome sequencing has the potential to assist conservation and management efforts by providing information on the taxonomic status and demographic history of populations and by estimating genome-wide levels of genetic variation associated with local adaptation and inbreeding (Allendorf et al. 2010; Steiner et al. 2013). Our work presents the first complete NWR genomes, representing the current gene pool of this subspecies. These genomes provided estimates of genome-wide levels of genetic diversity and inbreeding that may inform decisions on the genetic value of cells to be used in genetic rescue and assisted reproduction efforts. We also examined the recent population history and demography of these two white rhinoceros subspecies and identified potential regions of selection in the NWR that may suggest local adaptation and divergent evolution from the SWR. Because of the comparative approach we employed using two closely related populations, this work may be the first to use genome-wide analysis as an indicator of the recovery potential of an endangered species, which is relevant for evaluating extinction risk and conservation recovery strategies (Frankham et al. 2010).

Information on the taxonomic relationship, population structure, and divergence time between the NWR and SWR is relevant for designing conservation strategies that will assist rescuing the NWR. Recent genetic evidence using complete mitochondrial genomes (Harley et al. 2016) suggests the NWR and SWR represent subspecies of white rhinoceros, while others have used morphological differences to support both populations as distinct species (Groves et al. 2010). Our genome-wide analyses found modest levels of genetic divergence between the NWR and SWR, even though these subspecies show distinct genetic structure. The level of genomic divergence between the NWR and SWR appears consistent with other mammalian subspecies such as chimpanzees (0.0019) and gorillas (0.0016) (Prado-Martinez et al. 2013).

Estimates from demographic analyses suggest that these two subspecies diverged between 10 and 80 kya, with little or no recent gene flow. Population divergence times estimates differ depending on the method used (Zhou and Teo 2015); therefore, the variation in divergent estimates may be due to the fact that ∂a∂i infers split time from a population's allele frequency spectrum, while PSMC makes inferences based on the local density of heterozygotes across the genome. The more recent history of the southern white rhinoceros, such as the large bottleneck in the last century (Saragusty et al. 2016), could not be detected by using PSMC, presumably because there are too few recombination events in the genome to detect these recent changes in population size (Li and Durbin 2011). It should also be noted that PSMC can confound population structure with changes in effective population size and is less accurate in estimating those changes in the recent or very ancient past (Li and Durbin 2011).

The north/south split in population structure shown between the NWR and SWR is similar to the pattern observed among many other African ungulates (Lorenzen et al. 2012), thought to be driven by pluvial cycles. Lorenzen et al. (2012) found that for those species whose ranges extended into southern Africa, all but buffalo were structured into subspecies north and south of the equatorial forest belt. This suggests that a vicariance event such as the expansion of tropical forests across Central Africa during glacial cycles could have driven the common north/south divergence seen in many taxa.

The relatively close genetic relationship of the two white rhinoceros subspecies is promising for the potential success of reproductive attempts in rescuing the NWR by using the SWR as model population for physiological studies and surrogate females for embryo implantation. The genetic diversity represented in the NWR preserved cell lines at the San Diego Zoo Frozen Zoo is comparable to that of the SWRs, with higher levels of genome-wide heterozygosity and slightly lower levels of autozygosity in the NWR, consistent with the fact that the SWR has undergone a population bottleneck in the last century. The SWR population is thought to have been as low as 20–50 individuals at the beginning of the 20th century, and it has relatively recently recovered within the last 50 yr (Emslie 2012). These years of low population size likely led to the increased inbreeding and reduced genetic diversity seen in this study. In contrast, the decline of the NWR population happened relatively recently, with the population thought to be around 2360 in 1960 (Emslie and Brooks 1999) but has rapidly declined since then due to intense poaching (Emslie 2012).

The NWR samples analyzed in this study likely represent individuals from generations immediately after the recent population decline, before drift and inbreeding could reduce genetic diversity. The SWR samples likely represent the recent expansion of the population, as shown by the increased levels of inbreeding found in these individuals. The SWR samples show long tracts of homozygosity compared with that of the NWR, consistent with recent inbreeding. However, overall levels of autozygosity in both subspecies are low compared with other inbred species, such as the mountain gorilla (Xue et al. 2015) and Scandinavian wolves (Kardos et al. 2018), suggesting that inbreeding occurred recently and over relatively few generations. Genetic estimates of ROH will be important to any future rescue program for the NWR, as whole-genome sequencing provides a better estimate of inbreeding than pedigree analysis (Kardos et al. 2018).

To identify potentially adaptive trends between white rhinoceros subspecies, we identified a set of 28 genes as likely under selection in the NWR. Among the genes, several are associated with olfaction or smell perception, which have been found to quickly evolve during speciation in other mammalian species, including primates and pandas (Moyle 2005; Zhao et al. 2013; Paudel et al. 2015). It is possible that these regions have been under recent selection or soft selective sweep in the NWR and represent genomic areas of potential adaptive variation (Messer and Petrov 2013). These results are preliminary, but rapidly evolving olfactory receptors may be linked to any differences in ecology and behavior. While Groves et al. (2010) concluded that reported behavioral and ecological observations do not provide a clear distinction between the two subspecies, our results point to regions of the genome where further investigation is warranted.

We believe that the current NWR genetic material banked at the San Diego Zoo Frozen Zoo in the form of cell lines is promising for future genetic rescue efforts in saving this subspecies from extinction. This approach would involve the use of methods such as the artificial production of gametes by directed differentiation of pluripotent stem cells (PSCs) in vitro, or combined with maturation in vivo into germline stem/progenitor cells (Nayernia et al. 2006; Hayashi and Saitou 2013; Easley et al. 2015; Hendriks et al. 2015), and cloning by nuclear transfer of cryopreserved material (Saragusty et al. 2016). The possibility of generating artificial gametes from PSCs in mice has been demonstrated with fertile offspring born from gametes generated this way (Nayernia et al. 2006; Hayashi et al. 2011; Zhu et al. 2012; Hayashi and Saitou 2013), and further experiments indicate that gametes could be generated this way in other species (Aflatoonian et al. 2009; Eguizabal et al. 2011; Panula et al. 2011).

Interspecies somatic cell nuclear transfer can also be used to clone endangered species, which has been demonstrated, for example, in the wild ox, *Bos gaurus* (Lanza et al. 2000), and an extinct wild goat subspecies, *Capra pyrenaica pyrenaica* (Folch et al. 2009). Somatic nuclear transfer has also been suggested as a way to save other endangered wildlife populations in which the genetic diversity is thought to be insufficient for survival, such as the black-footed ferret (Wisely et al. 2015).

Other approaches have also been considered for genetic rescue of the NWR, including inter-crossing SWR with NWR from the wild (Emslie 2012) or from cryopreserved gametes (Saragusty et al. 2016). However, the single known hybrid between northern and southern white rhinoceroses was Nasi, born 1977 and died in 2007. This individual reached adulthood but never reproduced and was in relatively poor health (Groves et al. 2010). Nothing in our findings would invalidate the interbreeding of NWR and SWR; however, given the death of the one remaining male NWR and the limited number of NWR cryopreserved gametes, the amount of NWR genetic variation that could be maintained through interbreeding is likely limited. While some might argue against efforts to genetically rescue the NWR subspecies given the close evolutionary relationship with the SWR, this makes using the SWR as a surrogate more likely to succeed, and the benefits and lessons of any genetic rescue effort could apply to other rhinoceros species, as well as to mammalian species with similar life history and conservation concerns.

The NWR is extinct in the wild, and the two remaining female rhinoceroses cannot reproduce naturally. Previous investigators have suggested that the best hope to save this subspecies rests in the use of a genetic rescue approach taking advantage of the genetic lineages preserved in the San Diego Zoo Frozen Zoo (Saragusty et al. 2016). Here we have shown the genetic diversity represented by these preserved cell lines is comparable with that of the SWR samples used in this study. At the beginning of the last century, the SWR teetered on the brink of extinction, but through dedicated conservation efforts was able to recover and is now the largest population of rhinoceros in the world. The potential is there for the NWR to make a similar recovery. The newly sequenced genomes could allow a genetic rescue program to maximize retention of genetic diversity and minimize inbreeding. While technological and logistical hurdles certainly remain, this work shows that time may not have run out on the NWR.

## Methods

### Samples

We selected a total of 13 wild-born white rhinoceros samples, four SWR and nine NWR, none of which were related according to the known pedigree (Supplemental Fig. S1). Eight NWR samples were derived from cell lines preserved in the San Diego Zoo Frozen Zoo, and one sample corresponds to a zoo-based individual for which no viable frozen cells exist and only DNA were available. Of the NWR samples, seven individuals were from Sudan and two from Uganda (Christman 2011). All white rhinoceros cell lines were cultured, harvested, and chromosome banded following the techniques described by Houck et al. (1995). One individual had a diploid (2*n*) chromosome number of 81, a known variant in white rhinoceroses of the most common 2*n* = 82 karyotype (Houck et al. 1994). Utilization of samples was compliant with applicable regulatory procedures for CITES and the US Endangered Species Act. DNA was extracted using the DNeasy cell line kits (Qiagen) according to the manufacturer's instructions.

### Sequencing

Nine NWR and four SWR were sequenced by Cofactor Genomics using Illumina paired-end sequencing to a coverage of 10×–15×. Briefly, Genomic DNA libraries were constructed by shearing genomic DNA to the desired size using the Covaris S2 (Covaris). Following shearing, DNA was end-repaired and A-tailed to prepare for adaptor ligation. Indexed adaptors were ligated to sample DNA, and the adaptor-ligated DNA was then size-selected on a 2% SizeSelect E-Gel (Invitrogen) and amplified by PCR. Library quality was assessed by measuring nanomolar concentration and the fragment size in base pairs. Cluster generation and the following sequencing were performed according to the cluster generation manual and sequencing manual from Illumina (https://support.illumina.com/content/dam/illumina-support/documents/ documentation/system_documentation/cluster_station/ClusterStation_UserGuide_15018818_D.pdf). Base calls were generated using CASAVA 1.8.2 (Illumina), and the resulting demultiplexed sequence reads were filtered for low quality.

All samples were aligned to the SWR reference genome (cerSim1; accession ID: GCA 0002831551.1) using SAMtools mpileup (Li et al. 2009), and variants were called using the multiallelic calling mode in BCFtools (bcftools m). Variants were filtered using the following criteria:

- variants within 3 bp of an indel;
- clusters of indels separated by ≤10 bp, allowing only one to pass;
- quality score <10;
- Mann-Whitney *U* <0.1 and quality <15; and
- ancestral count <2 and quality <15.

In order to identify scaffolds in the rhinoceros genome corresponding to the X Chromosome, we first attempted to BLAST all scaffolds against the horse X Chromosome. However, a large number of scaffolds contained sequences highly similar to the horse X (see Supplemental Table S5) and were subsequently included in the analysis.

### Population structure and phylogeny

We used the software ADMIXTURE (Alexander et al. 2009) to detect population structure and levels of admixture between the northern and southern subspecies. We used a set of approximately 144,000 SNPs that were thinned for potential linkage disequilibrium using the software PLINK v1.9 (Gaunt et al. 2007; Chang et al. 2015). Specifically, we used a 50-SNP sliding window, advanced 10 SNPs at a time, and removed any SNPs with and *R*^2^ value of >0.1. We used this data set to perform 10-fold cross validation using K values ranging from one to five. The same set of markers were used to perform PCA using EIGENSTRAT (Price et al. 2006), which corrects for population stratification. These markers were further used to generate a maximum likelihood tree using SNPhylo (Lee et al. 2014) and 100 nonparametric bootstrap samples.

### Demographic history and divergence time

PSMC (Li and Durbin 2011) was used to estimate historical population size and divergence of the NWR and SWR. Here, we used a generation time of 8 yr and mutation rate of 2.5 × 10^−8^ (substitutions/site/yr) (Hillman-Smith et al. 1986). In order to account for low genome coverage, we down-sampled our 15× genome to 10× and found a nearly identical loss of heterogeneity to that of the horse genome. We therefore used a false-negative rate of 0.06 for our 15× rhinoceros genomes used in the analysis, as previously described (Orlando et al. 2013).

We also estimate demographic history using ∂a∂i by testing a series of four increasingly complex models (Gutenkunst et al. 2009). We selected the four NWR with the highest coverage and, along with the four SWR, calculated the site-frequency spectrum (Fig. 3B). The simplest model consisted of a split into two populations, with no growth or migration between populations, and the complex model consisted of a split into two populations, with change in size from past to present and migration between populations. A mutation rate of 2.5 × 10^−8^ was also used for inferring scaled population parameters. Likelihood and parameter estimates for each model can be found in the Supplemental Materials.

Levels of inbreeding within individuals in the northern and southern subspecies were evaluated by analyzing genome-wide ROH. To identify ROH, we calculated genome-wide heterozygosity in overlapping regions of 1 Mbp with 200-kbp sliding windows. We plotted the density of heterozygosity in each window, which showed regions of low heterozygosity in both the SWR and NWR. By using previous estimates from the literature (Prado-Martinez et al. 2013) and visualizing the data using density plots, we used a threshold value of 0.0004, or 400 per 1 Mbp, to determine the window that could be considered a ROH. We then calculated the percentage of the genome that could be considered autozygous (*F*_roh_). The length distribution of ROH was calculated using R (R Core Team 2016). The length is defined as the number of consecutive, overlapping 1-Mbp ROH, determined as described above.

### Gene annotation

RNA-seq data were generated from brain, testis, oviduct, and fibroblast cells using paired-end reads in the Illumina MiSeq platform. We lifted the Ensembl 85 (Yates et al. 2016) release annotations for both cow and dog to the rhinoceros assembly using a progressive Cactus alignment (Paten et al. 2011). We then fed the protein coding transcripts as hints to AUGUSTUS (Stanke et al. 2006) along with RNA-seq data on a per-transcript basis. The output of this underwent a consensus finding tool with the original mapped transcripts that performs filtering and determines if we have a high-quality ortholog in any of our input sets (Fiddes et al. 2018).

### Selection

We calculated Watterson's estimate of θ and θπ for nonoverlapping windows of 50 kb in our nine NWR genomes and calculated Tajima's *D* for each window. We then selected the regions in the outlying 1% quantile using R (R Core Team 2016), representing regions potentially under balancing or positive selection. We used the annotation available for the white rhinoceros on the UCSC genome browser (cerSim1) to identify all genes occurring in these outlier regions. We used biomaRt (Durinck et al. 2005, 2009) to identify the HGNC code and Gene Ontology for all genes that contained SNPs that fall within transcribed regions. Specific enrichment for a particular class of genes was identified using PANTHER (Mi et al. 2013).

## Data access

The data generated from this study have been submitted to the NCBI BioProject (https://www.ncbi.nlm.nih.gov/bioproject/394025) under accession number PRJNA394025.

## Supplementary Material

Supplemental Material
