# Supplementary material for: Evaluating recovery potential of the northern white rhinoceros from cryopreserved somatic cells
Source: Genome Res. 2018 Jun;28(6):780–8. doi: 10.1101/gr.227603.117 (PMC5991516; doi:10.1101/gr.227603.117)
Supplement: Supplemental Material [file supp_28_6_780__index.html]

Evaluating recovery potential of the northern white rhinoceros from cryopreserved somatic cells — Evaluating recovery potential of the northern white rhinoceros from cryopreserved somatic cells — Supplemental Material 

# Evaluating recovery potential of the northern white rhinoceros from cryopreserved somatic cells

## Supplemental Material

- Supplemental\_Material.pdf
