# Supplementary material for: Evaluating recovery potential of the northern white rhinoceros from cryopreserved somatic cells
Source: Genome Res. 2018 Jun;28(6):780–8. doi: 10.1101/gr.227603.117 (PMC5991516; doi:10.1101/gr.227603.117)
Supplement: Supplemental Material [file supp_gr.227603.117_Supplemental_Material.pdf]

Contents

1 Northern white rhinoceros pedigree 1

2 Genetic Divergence 2

3 Shared SNP Polymorphism 2

4 Admixture and PCA 4

5 Mitochondrial Tree 5

6 Demographic Inference Using ∂a∂i 7

7 Inbreeding 8

8 Selection 9

9 Identification of the X chromosome 14

1 Northern white rhinoceros pedigree

Figure 1: NWR pedigree highlighting individuals sequenced in this study (in blue box) presumably unrelated, with name, studbook number, ID number, and ploidy number

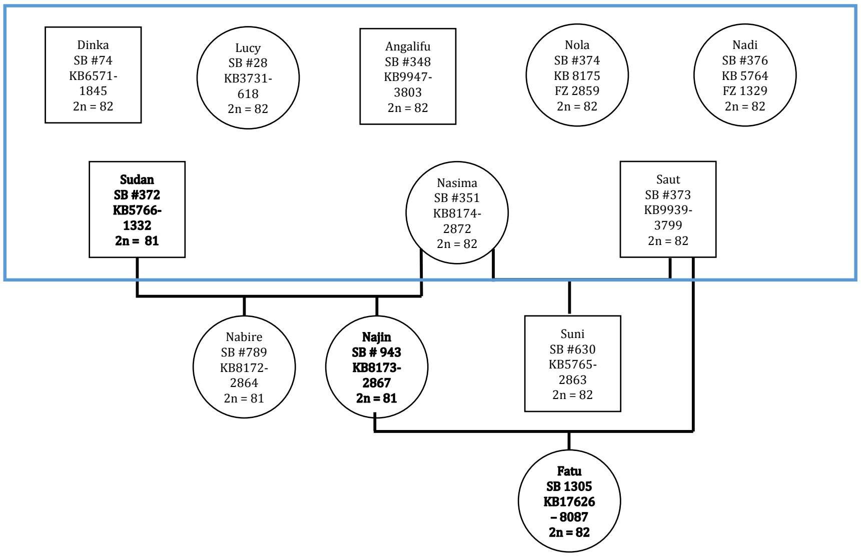

2 Genetic Divergence

Pair-wise genetic divergence was estimated between all pairs of individuals using sites callable among all individuals, and defined as (2*homs+hets)/(2*callable fraction of genome), as defined in (Prado-Martinez et al., 2013). Divergence values for all individuals are shown in Supplementary Material Table 1. Calculations were performed on the full set of 9.4 million SNPs.

3 Shared SNP Polymorphism

In order to calculate shared polymorphism between the NWR and SWR, we took the average polymorphism of all possible combinations of the nine NWR

Table 1: Pair-wise genetic divergence for all rhinoceroses included in this study

	SB 28	SB 377	SB 376	SB 372	SB 156	SB 74	SB 24	SB 147	SB 351	SB 374	SB 373	SB 348	SB 34
SB 28	0	0	0	0	0	0	0	0	0	0	0	0	0
SB 377	0.0020231648	0	0	0	0	0	0	0	0	0	0	0	0
SB 376	0.0021290369	0.0019646831	0	0	0	0	0	0	0	0	0	0	0
SB 372	0.001257861	0.0020856797	0.002162311	0	0	0	0	0	0	0	0	0	0
SB 156	0.0040196095	0.0041148944	0.0041511934	0.0043498295	0	0	0	0	0	0	0	0	0
SB 74	0.0026236107	0.0027471281	0.0027456156	0.0026165525	0.0047556725	0	0	0	0	0	0	0	0
SB 24	0.0037181262	0.0040518753	0.0041189276	0.0040216261	0.001726723	0.0046316509	0	0	0	0	0	0	0
SB 147	0.0039747399	0.0041814425	0.0042232872	0.0042802565	0.0015563194	0.0048781816	0.0009972141	0	0	0	0	0	0
SB 351	0.0019666997	0.0021219787	0.0020624887	0.0015658983	0.0048459158	0.0026200816	0.0043538628	0.0045887979	0	0	0	0	0
SB 374	0.0016384963	0.0021108874	0.002162311	0.0012255952	0.0044022614	0.0026654554	0.0039858313	0.0042484949	0.0012855894	0	0	0	0
SB 373	0.0020266939	0.0021542445	0.0021184497	0.0016516043	0.0048297829	0.0026468017	0.004334705	0.0045938395	0.0008686551	0.0013445753	0	0	0
SB 348	0.0020372811	0.0019384672	0.0019152761	0.0020448434	0.0041264899	0.002737045	0.0040841411	0.0041905173	0.0020246773	0.0020473641	0.0020947545	0	0
SB 34	0.0040322133	0.004169847	0.0041900131	0.004374533	0.0018164622	0.0046598835	0.001855282	0.0018320909	0.0047566808	0.0043760455	0.0047460936	0.0041920297	0

Table 2: Unique number of SNPs estimated for the NWR and SWR individuals studied. Unique number of SNPs are SNPs that only occur in one individual

Subspecies	ID	Number of Unique SNPs
SWR	SB 34	102537
SWR	SB 24	106103
SWR	SB 147	124685
SWR	SB 156	97415
NWR	SB 28	118155
NWR	SB 377	82313
NWR	SB 376	72136
NWR	SB 372	54443
NWR	SB 74	97750
NWR	SB 351	56155
NWR	SB 374	24510
NWR	SB 373	79715
NWR	SB 348	75545

individuals rarefied down to four individual samples. We then compared this to the four SWR individuals, and determined how many SNPs were polymophic in both populations, only polymorphic in one population, and how many were fixed differences between the two populations. Calculations were done on the full set of SNPs.

Figure 2: Venn diagram representing shared polymorphism in the NWR and SWR. Colored regions represent SNP loci polymorphic in each population, overlapping area represents loci polymorphic in both samples. Outside shaded area represents the number of loci with fixed differences.

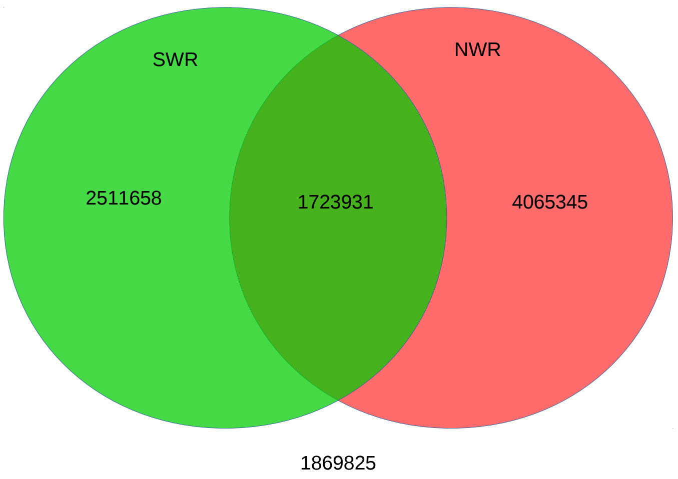

4 Admixture and PCA

After performing 10-fold cross validation on the ADMIXTURE dataset, we found little difference between values of K=1 and K=2 (0.73 and 0.74 respectively), likely due to the recent separation of the two subspecies.

Figure 3: 10-fold cross-validation validation as performed in ADMIXTURE, using K values from 1 to 5 and a dataset of approximately 144,000 SNPs. Y-axis is the cross-validation error, and the x-axis is the K value.

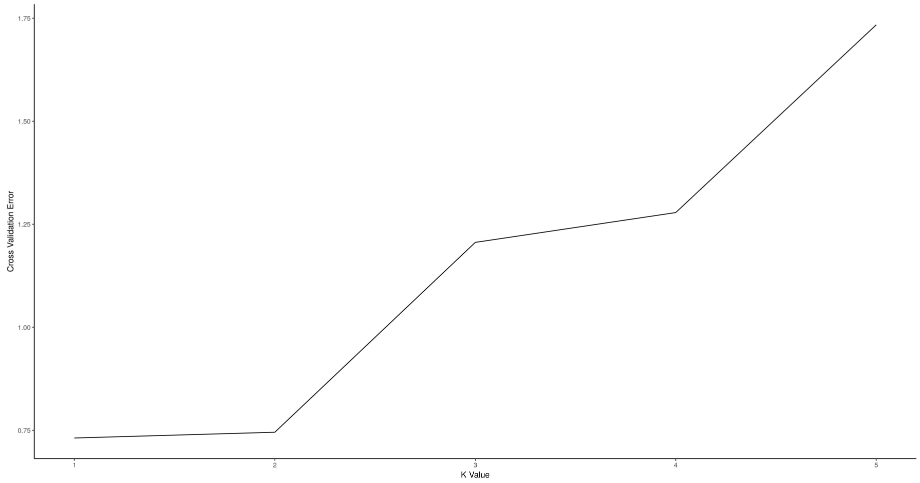

5 Mitochondrial Tree

The final mitochondrial alignment included nine northern and five southern white rhinoceroses, four of the southern sequences obtained from whole genome sequencing and one from Genbank (accession number NC_001808). The control region was excluded from the alignment. Phylogenetic analyses in BEAST 1.6.1 (Drummond and Rambaut, 2007) were performed considering a single partition with a model of sequence evolution corresponding to HKY + G + I, and five partitions as follow: tRNAs, rRNAs, and first, second and third codon sites of the protein coding genes. jModelTest 0.1 (Posada 2008) was used to select models of sequence evolution according to the Akaike Information Criterion: GTR + I (first and third codons, and rRNAs) and TrN (second codon, tRNAs). The monophyly of southern and northern white rhinoceroses was constrained according to a tree inferred using MrBayes 3.1.2 (Ronquist and Huelsenbeck, 2003). The Bayesian inference consisted of two concurrent runs with four Markov chains

(one cold and seven heated chains with a temperature of 0.2), twenty million generations (sampled every 1,000 generations), and a 10% burn-in. We verified that potential scale reduction factors were near to 1.0 for all parameters, and that the average standard deviation of split frequencies was below 0.01. We visualized convergence of runs to stationarity using Tracer version 1.6 (Drummond and Rambaut, 2007) by verifying no trends in generation versus logL plots. To estimate the time to the most recent common ancestor (tMRCA) of both rhino populations, Beast analyses were performed assuming a constant population size as tree prior and strict molecular clock with a rate of evolution (mean number of substitutions per site per unit of time) corresponding to 0.0052 (Steiner et al. in review). The run was performed 108 MCMC generations, with samples taken every 104 steps, and the first 5 x 104 steps removed as burn-in. Tracer (Drummond and Rambaut, 2007) was employed to analyze the autocorrelation tree and effective sample size for parameter estimates. The final tree was estimated in TreeAnnotator v1.8.2 and visualized in FigTree v. 1.4.2.

Species tree inference estimated the time to the most recent common ancestor of both white rhinoceros populations around 720 kya (575–862 kya). For the SWR and NWR populations, the age of the most recent common ancestor was calculated to 26 kya (8–48 kya) and 13 kya (4–25 kya), respectively, suggesting a relatively recent origin of mitochondrial haplotypes for both subspecies at the end of the Pleistocene. This is consistent with a previous work on complete white rhinoceros mitochondrial genomes showing the NWR and SWR as distinct monophyletic clades diverging between 0.46 and 0.97 million years ago using Bayesian inferences (Harley et al., 2016).

Our species tree inference estimated the mitochondrial divergence time around 720 kya for the two rhino subspecies. As noted above, estimates from both $\partial a \partial i$ and PSMC suggest that these two subspecies diverged less than 80 kya. This

large difference in divergence times could at partially explained by the fact that both ∂a∂i and PSMC estimate population divergence, and our estimates from the mitochondrial data are for the most recent common ancestor of the two mitochondrial haplotypes, which must occur later than the time of population divergence.

Figure 4: Mitochondrial tree as generated through BEAST

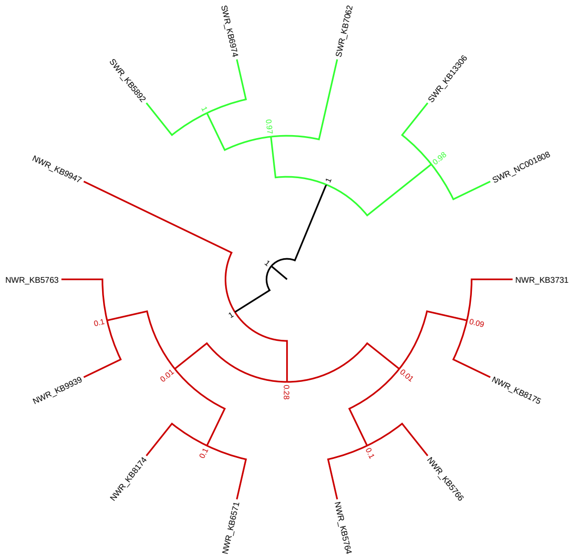

6 Demographic Inference Using ∂a∂i

For the ∂a∂i analysis, we used 8.7 million SNPs callable in all four southern white rhinoceroses and four of the northern white rhinoceroses. We used the folded frequency spectrum, which considers only minor allele frequencies. We fit to the data a series of increasing complexity models. One model included a split into two populations, followed by exponential growth. The second set of models constrained the northern and southern population sizes to fractions of the ancestral population size, followed by either exponential growth or a growth model similar to that used by (Gutenkunst et al., 2009). Results are presented

in Supplemental Table 3. We include both the estimates scaled to θ, as reported by ∂a∂i, as well as estimates in natural units when appropriate.

Table 3: Results from the three tested ∂a∂i models. Tsplit represents estimated split from ancestral population; Na is ancestral population size; nu1 and n1 the is size of the NWR and SWR populations at the time of the split from the ancestral population; for the fractional models, s is the fraction of the ancestral population which becomes the NWR at the split, mNS and mSN is the north-south and south-north migration rate.

	likelihood	theta	nu1	nu2	nu1F	nu2F	mNS	mSN	T_split
Split Model	-8784860.10	3006281.45	216552.44	253.38	4912.69	3486.96	1975.91	2351.05	193799.45
	-881633.70	3614580.14	2617626.71	4430.95	706.14	1783.89	13905.41	85923.32	20311.46
	-874028.40	3695210.99	3275055.85	856.60	370.15	3940.39	0.05	42511.52	11006.33
	-872445.30	3716924.37	4341.20	470.51	1803.29	9233.60	54.96	18550.98	9212.84
	-872045.05	3751853.99	757.49	247.38	6655.04	19869.33	0.00	0.00	6450.83
	likelihood	theta	s		nu1F	nu2F	m12	m21	T_split
Fraction Split 1	-905670.36	3677343.26	0.53		1701.27	971.97	6.48	5.30	16806.09
	-887171.97	3665656.89	0.18		3273.10	703.94	10.09	38728.90	15626.93
	-879198.41	3619272.14	0.85		1757.10	1677.22	0.46	51844.01	18107.08
	-878928.75	3667540.11	0.97		930.72	3696.27	16.59	164.91	11198.29
	-875869.26	3633939.62	0.93		1391.48	2330.23	2.72	38574.67	15296.77
	-874778.16	3640720.90	0.95		1261.68	2837.48	1.20	32681.38	14227.77
Fractional Split 2	-884651.45	3623150.22	0.54		2229.53	1060.15	3521.04	49103.49	18998.98
	-878410.04	3694735.70	0.98		754.35	6774.81	153.91	162.97	9465.15
	-876508.28	3635435.92	0.93		1417.03	2742.03	7869.74	33841.42	15726.68
	-876072.12	3628897.44	0.92		1437.98	2306.38	92.86	38653.13	15692.17
	-875921.45	3631756.19	0.93		1408.87	2336.97	116.09	37958.55	15442.96

7 Inbreeding

We calculated the number of regions that could be considered a run of homozygosity (ROH), which is considered a good measure of inbreeding (McQuillan et al., 2008). We choose a window size of 1 Mbp according to (Pemberton et al., 2012), which identified regions of homozygosity smaller than 0.5 Mbp as the result of background relatedness, and regions larger than 1.6 Mbp as the result of recent parental relatedness.

We determined shared runs of homozygosity by calculating ROH shared by two or more rhinoceroses in each population. To compare the NWR to the SWR, we resampled all possible four rhinoceroses combinations from the nine NWR, and determined how often a ROH was shared by two or more rhinoceroses in a 4 individual sample

Supplementary Material Figure 5. Runs of homozygosity shared by two or more individuals in each population, for lengths of 1, 5, 10, 15, 20, 25, and 30 Mbp. Error bars represent the standard deviation from resampling all possible four rhinoceroses combinations in the NWR.

Figure 5: Runs of homozygosity shared by two or more individuals in each population, for lengths of 1, 5, 10, 15, 20, 25, and 30 Mbp. Error bars represent the standard deviation from resampling all possible four rhinoceroses combinations in the NWR

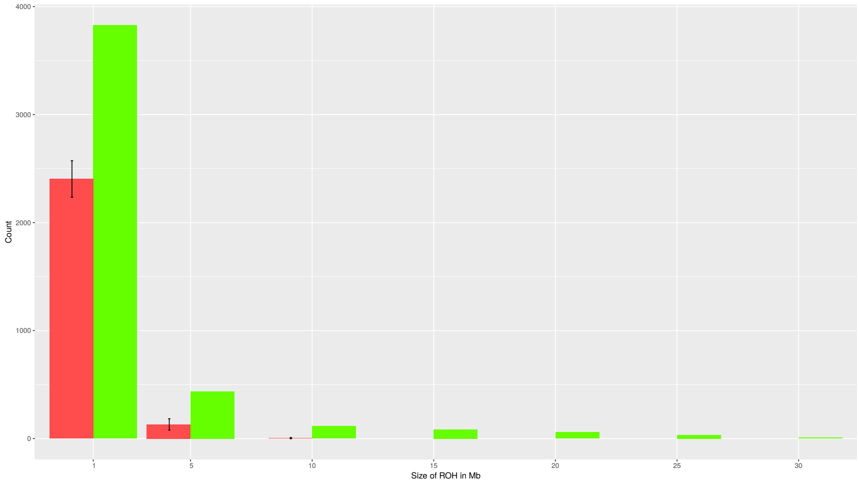

8 Selection

Figure 6: Tajima's D values for all scaffolds in the southern white rhinoceros genome. Red lines represent the boundaries of the 1% quantiles.

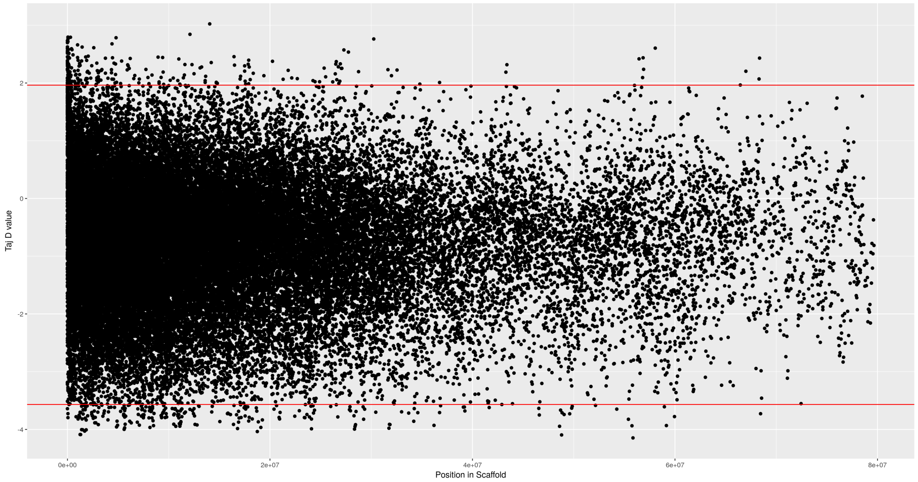

Table 4: List of all genes containing outliers coding SNPs as identified by the Tajimas D test.

Gene description	Ensembl ID	SNP count	HGNC symbol	Gene Ontology ID
interferon beta 1	ENSCAFG00000001653	19	IFNB1	GO:0002250, GO:0002281, GO:0002286, GO:0002312, GO:0002323, GO:0005125, GO:0005126, GO:0005132, GO:0005576, GO:0005615, GO:0006952, GO:0006959, GO:0007166, GO:0007596, GO:0008811, GO:0009615, GO:0030101, GO:0030183, GO:0033141, GO:0035458, GO:0042100, GO:0042742, GO:0043330, GO:0045071, GO:0045089, GO:0045343, GO:0045581, GO:0045944, GO:0051607, GO:0060337, GO:0060338, GO:0071359, GO:0071360, GO:0071549, GO:0098586, GO:2000552, GO:2001235
protease, serine 58	ENSCAFG00000003823	31	PRSS58	GO:0004252, GO:0005576, GO:0006508, GO:0008233, GO:0008236, GO:0016787
potassium channel tetramerization domain containing 12	ENSCAFG00000005068	1	KCTD12	GO:0003723, GO:0005886, GO:0016020, GO:0030054, GO:0042734, GO:0042802, GO:0045202, GO:0045211, GO:0051260, GO:0070062

F-box and leucine rich repeat protein 3	ENSCAFG00000005072	1	FBXL3	GO:0000151, GO:0000209, GO:0004842, GO:0005515, GO:0005634, GO:0005737, GO:0005829, GO:0016567, GO:0016604, GO:0019005, GO:0031146, GO:0031648, GO:0042752, GO:0043153, GO:0043161, GO:0043687, GO:0048511
ER membrane protein complex subunit 3	ENSCAFG00000005202	24	EMC3	GO:0003674, GO:0008150, GO:0016020, GO:0016021, GO:0034975, GO:0072546
olfactory receptor family 4 subfamily E member 2	ENSCAFG00000005679	2	OR4E2	GO:0004871, GO:0004888, GO:0004930, GO:0004984, GO:0005886, GO:0007165, GO:0007186, GO:0007608, GO:0016020, GO:0016021, GO:0050896, GO:0050907, GO:0050911
olfactory receptor family 52 subfamily N member 2	ENSCAFG00000006321	2	OR52N2	GO:0004871, GO:0004930, GO:0004984, GO:0005886, GO:0007165, GO:0007186, GO:0007608, GO:0016020, GO:0016021, GO:0050896, GO:0050911
heat shock protein family H (Hsp110) member 1	ENSCAFG00000006538	14	HSPH1	GO:0000166, GO:0000774, GO:0005515, GO:0005524, GO:0005576, GO:0005634, GO:0005654, GO:0005737, GO:0005829, GO:0005874, GO:0006898, GO:0006986, GO:0043014, GO:0043234, GO:0045345, GO:0045944, GO:0051085, GO:0051135, GO:0061098, GO:0070062, GO:0071682, GO:1900034, GO:1903748, GO:1903751, GO:1903753, GO:2001234
olfactory receptor family 10 subfamily V member 1	ENSCAFG00000007561	1	OR10V1	GO:0004871, GO:0004930, GO:0004984, GO:0005549, GO:0005886, GO:0007165, GO:0007186, GO:0007608, GO:0016020, GO:0016021, GO:0050896, GO:0050911
olfactory receptor family 5 subfamily G member 3	ENSCAFG00000007988	8	OR5G3	
ADP-ribosyltransferase 3	ENSCAFG00000008589	4	ART3	GO:0003950, GO:0003956, GO:0005576, GO:0005886, GO:0005887, GO:0006471, GO:0006501, GO:0016020, GO:0016740, GO:0016757, GO:0031225, GO:0070062

NADH:ubiquinone oxidoreductase subunit B4	ENSCAFG00000011204	2	NDUFB4	GO:0005739, GO:0005743, GO:0005747, GO:0006120, GO:0006979, GO:0008137, GO:0016020, GO:0016021, GO:0031965, GO:0032981, GO:0055114, GO:0070062, GO:0070469
olfactory receptor family 2 subfamily W member 1	ENSCAFG00000012133	17	OR2W1	GO:0004871, GO:0004930, GO:0004984, GO:0005886, GO:0007165, GO:0007186, GO:0007608, GO:0016020, GO:0016021, GO:0050896, GO:0050911
galectin 12	ENSCAFG00000015091	6	LGALS12	GO:0005634, GO:0005739, GO:0006915, GO:0030246, GO:0030395, GO:0045598, GO:0050994, GO:0097193
acyl-CoA synthetase medium chain family member 3	ENSCAFG00000017952	15	ACSM3	GO:0000166, GO:0003674, GO:0003824, GO:0003996, GO:0004321, GO:0005524, GO:0005575, GO:0005739, GO:0005759, GO:0006629, GO:0006631, GO:0006633, GO:0006637, GO:0008152, GO:0008217, GO:0015645, GO:0016874, GO:0042632, GO:0046872, GO:0047760
alpha-2-macroglobulin	ENSCAFG00000025567	4	A2M	GO:0001869, GO:0002020, GO:0002576, GO:0004866, GO:0004867, GO:0005096, GO:0005102, GO:0005515, GO:0005576, GO:0005615, GO:0005829, GO:0007597, GO:0010466, GO:0010951, GO:0019838, GO:0019899, GO:0019959, GO:0019966, GO:0022617, GO:0030414, GO:0031093, GO:0043120, GO:0043547, GO:0048306, GO:0048863, GO:0051056, GO:0070062, GO:0072562
olfactory receptor family 8 subfamily K member 1	ENSCAFG00000028823	64	OR8K1	GO:0004871, GO:0004930, GO:0004984, GO:0005549, GO:0005886, GO:0007165, GO:0007186, GO:0007608, GO:0016020, GO:0016021, GO:0050896, GO:0050911

immunoglobulin heavy constant mu	ENSCAFG00000030258	15	IGHM	GO:0002250, GO:0002376, GO:0003697, GO:0003823, GO:0005515, GO:0005576, GO:0005615, GO:0005886, GO:0006910, GO:0006911, GO:0006958, GO:0009897, GO:0009986, GO:0016020, GO:0016021, GO:0019731, GO:0031210, GO:0034987, GO:0042834, GO:0045087, GO:0050829, GO:0050853, GO:0050871, GO:0050900, GO:0070062, GO:0071756, GO:0071757, GO:0072562
olfactory receptor family 5 subfamily M member 3	ENSCAFG00000032727	8	OR5M3	GO:0004871, GO:0004930, GO:0004984, GO:0005549, GO:0005886, GO:0007165, GO:0007186, GO:0007608, GO:0016020, GO:0016021, GO:0050896, GO:0050911
olfactory receptor family 56 subfamily A member 1	ENSBTAG00000000368	2	OR56A1	GO:0001591, GO:0001963, GO:0004871, GO:0004930, GO:0004984, GO:0005886, GO:0005887, GO:0007165, GO:0007186, GO:0007194, GO:0007195, GO:0007608, GO:0009636, GO:0014059, GO:0016020, GO:0016021, GO:0030672, GO:0035240, GO:0042493, GO:0043266, GO:0048148, GO:0048149, GO:0050896, GO:0050911, GO:0051481, GO:0051967, GO:0060158, GO:1901386
tumor protein D52 like 3	ENSBTAG00000011160	40	TPD52L3	GO:0005515
transmembrane protein 64	ENSBTAG00000011268	1	TMEM64	GO:0005783, GO:0016020, GO:0016021, GO:0043462, GO:0044339, GO:0045600, GO:0045668, GO:0045672, GO:0045780, GO:0051480, GO:0090090
phospholipid scramblase 4	ENSBTAG00000011986	3	PLSCR4	GO:0005509, GO:0005515, GO:0005886, GO:0016020, GO:0016021, GO:0017121, GO:0017124, GO:0017128, GO:0019899, GO:0042609, GO:0070062, GO:0071222

centrin 1	ENSBTAG00000012320	2	CETN1	GO:0000922, GO:0005509, GO:0005515, GO:0005737, GO:0005813, GO:0005814, GO:0005815, GO:0005856, GO:0007049, GO:0008017, GO:0031683, GO:0032391, GO:0032795, GO:0034605, GO:0046872, GO:0051301, GO:0005509, GO:0007214, GO:0008277, GO:0032228, GO:0046872, GO:0050966
olfactory receptor family 2 subfamily S member 2	ENSBTAG00000032670	47	OR2S2	GO:0004871, GO:0004930, GO:0004984, GO:0005886, GO:0007165, GO:0007186, GO:0007608, GO:0016020, GO:0016021, GO:0050896, GO:0050911
olfactory receptor family 2 subfamily D member 3	ENSBTAG00000038518	37	OR2D3	GO:0004871, GO:0004930, GO:0004984, GO:0005886, GO:0007165, GO:0007186, GO:0007608, GO:0016020, GO:0016021, GO:0050896, GO:0050911
G protein-coupled receptor 39	ENSBTAG00000047036	7	GPR39	GO:0004871, GO:0004930, GO:0005886, GO:0005887, GO:0007165, GO:0007186, GO:0016020, GO:0016021, GO:0046872
olfactory receptor family 56 subfamily B member 4	ENSBTAG00000047176	11	OR56B4	GO:0004871, GO:0004930, GO:0004984, GO:0005886, GO:0007165, GO:0007186, GO:0007608, GO:0016020, GO:0016021, GO:0050896, GO:0050911

9 Identification of the X chromosome

In order to identify scaffolds in the rhino genome corresponding to the X chromosome, we first attempted to BLAST all scaffolds against the horse X chromosome. However, a large number of scaffolds contained sequences highly similar to the horse X. We also determined the location of all genes occurring on the horse X chromosome using the UCSC Table Browser, and then identified the homologous genes in the rhino genome using biomaRt (Durinck et al., 2005) R (R Core Team, 2016). We identified 45 scaffolds in the white rhinoceros genome

with homologs of genes found in the horse X chromosome. These scaffolds represent 17% of the total size of the rhino genome. Due to difficulties in identifying X chromosome genomic regions in the rhino genomes, we did not filter or exclude the scaffolds identified as X chromosome in any of the genomic analyses. These results are shown in Table 5.

Table 5: Scaffolds in the southern white rhino genome with homologs on the horse X chromosome. The table contains the scaffold ID, the size of the scaffold, and the number of genes associated with the horse X chromosome found on the scaffold

Scaffold	Size	Count	Scaffold	Size	Count
JH767773	18099069	103	JH767905	395792	3
JH767774	17977685	76	JH767914	275042	3
JH767807	9651078	52	JH768001	39903	3
JH767790	12359428	45	JH767733	39176111	2
JH767876	1268962	43	JH767755	25004955	2
JH767813	8371206	38	JH767771	18387346	2
JH767823	5930997	24	JH767844	3258986	2
JH767846	3216515	23	JH767857	2430860	2
JH767870	1596305	20	JH767863	1968646	2
JH767785	14444393	17	JH767877	1169348	2
JH767819	7485276	15	JH767936	137788	2
JH767831	4435101	15	AKZM01054956	6131	1
JH767838	3782617	13	JH767732	44174171	1
JH767841	3625529	12	JH767739	33267342	1
JH767890	699797	11	JH767741	32302253	1
JH767897	472045	9	JH767745	29576853	1
JH767907	344087	7	JH767752	26277727	1
JH767882	860638	6	JH767791	12347721	1
JH767884	873626	6	JH767796	11665060	1
JH767866	1829894	5	JH767837	4025681	1
JH767910	334094	4	JH767899	463069	1
JH767852	2826735	3	JH767917	290250	1
JH767892	532191	3			

References

Drummond, A. J. and Rambaut, A., 2007. Beast: Bayesian evolutionary analysis by sampling trees. *BMC Evolutionary Biology*, **7**(1):214.

Durinck, S., Moreau, Y., Kasprzyk, A., Davis, S., De Moor, B., Brazma, A., and Huber, W., 2005. Biomart and bioconductor: a powerful link between biological databases and microarray data analysis. *Bioinformatics*, **21**(16):3439–3440.

Gutenkunst, R. N., Hernandez, R. D., Williamson, S. H., and Bustamante, C. D., 2009. Inferring the joint demographic history of multiple populations from multidimensional SNP frequency data. *PLOS Genetics*, **5**(10):1–11.

Harley, E. H., de Waal, M., Murray, S., and O'Ryan, C., 2016. Comparison of whole mitochondrial genome sequences of northern and southern white rhinoceroses (*Ceratotherium simum*): the conservation consequences of species definitions. *Conservation Genetics*, **17**(6):1285–1291.

McQuillan, R., Leutenegger, A.-L., Abdel-Rahman, R., Franklin, C. S., Pericic, M., Barac-Lauc, L., Smolej-Narancic, N., Janicijevic, B., Polasek, O., Tenesa, A., *et al.*, 2008. Runs of homozygosity in european populations. *The American Journal of Human Genetics*, **83**(3):359–372.

Pemberton, T. J., Absher, D., Feldman, M. W., Myers, R. M., Rosenberg, N. A., and Li, J. Z., 2012. Genomic patterns of homozygosity in worldwide human populations. *The American Journal of Human Genetics*, **91**(2):275–292.

Prado-Martinez, J., Sudmant, P. H., Kidd, J. M., Li, H., Kelley, J. L., Lorente-Galdos, B., Veeramah, K. R., Woerner, A. E., OConnor, T. D., Santpere, G., *et al.*, 2013. Great ape genetic diversity and population history. *Nature*, **499**(7459):471–475.

R Core Team, 2016. *R: A Language and Environment for Statistical Computing.* R Foundation for Statistical Computing, Vienna, Austria.

Ronquist, F. and Huelsenbeck, J. P., 2003. Mrbayes 3: Bayesian phylogenetic inference under mixed models. *Bioinformatics*, **19**(12):1572–1574.
